# Supplementary material for: SAM-DNMT3A, a strategy for induction of genome-wide DNA methylation, identifies DNA methylation as a vulnerability in ER-positive breast cancers
Source: Nat Commun. 2024 Dec 1;15:10449. doi: 10.1038/s41467-024-54824-8 (PMC11609277; doi:10.1038/s41467-024-54824-8)
Supplement: Supplementary file 1 — Supplementary Information [file 41467_2024_54824_MOESM1_ESM.pdf]

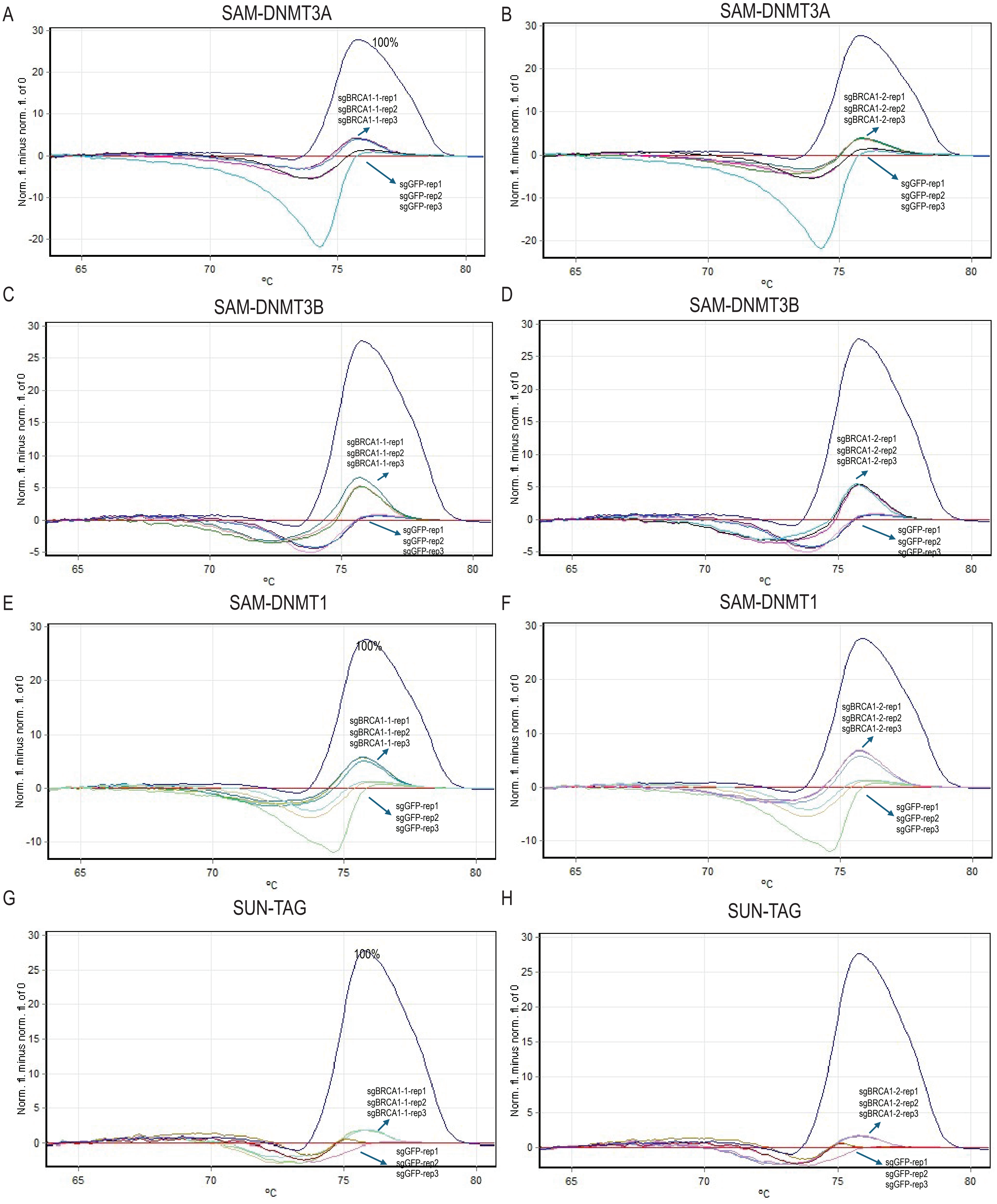


**Supplementary Figure 1: SAM-DNMT3A induces high levels of DNA methylation at desired sites.** (A,B) HRM profiles of SAM-DNMT3A induction of DNA methylation at the BRCA1 promoter. (C,D) HRM profiles of SAM-DNMT3B induction of DNA methylation at the BRCA1 promoter. (E,F) HRM profiles of SAM-DNMT1 induction of DNA methylation at the BRCA1 promoter. (G,H) HRM profiles of SunTag-DNMT3A induction of DNA methylation at the BRCA1 promoter.

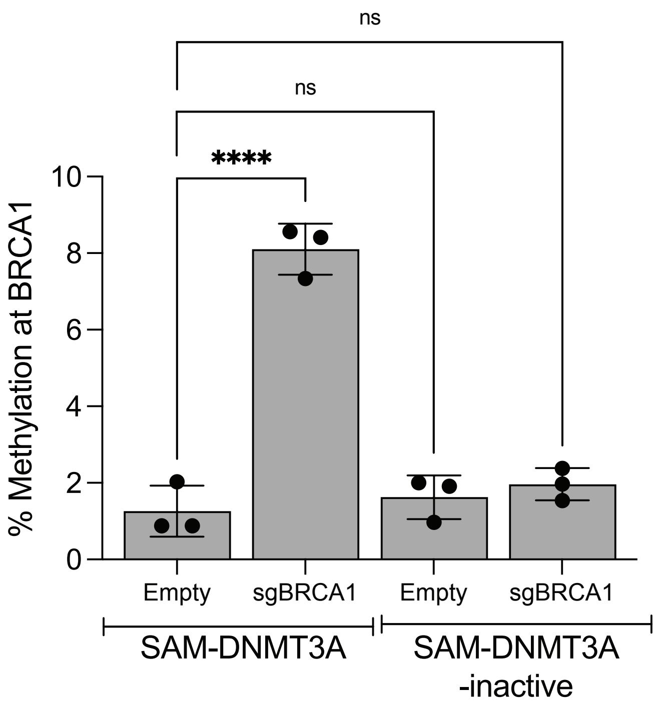


**Supplementary Figure 2: Catalytically inactive DNMT3A does not induce DNA methylation with the SAM-DNMT3A system.** HRM assay using BRCA1 probes in HEK293T cells expressing SAM-DNMT3A or SAM-DNMT3A-inactive with a BRCA1 targeting sgRNA. pValue was calculated using one way ANOVA. Data are mean +/-SD, n=3 biological replicates.
(P = 1.5e-6 (sgBRCA_1, SAM-DNMT3A), 0.38 (Empty, SAM-DNMT3A-inactive), 0.79 (sgBRCA1, SAM-DNMT3A-inactive)).

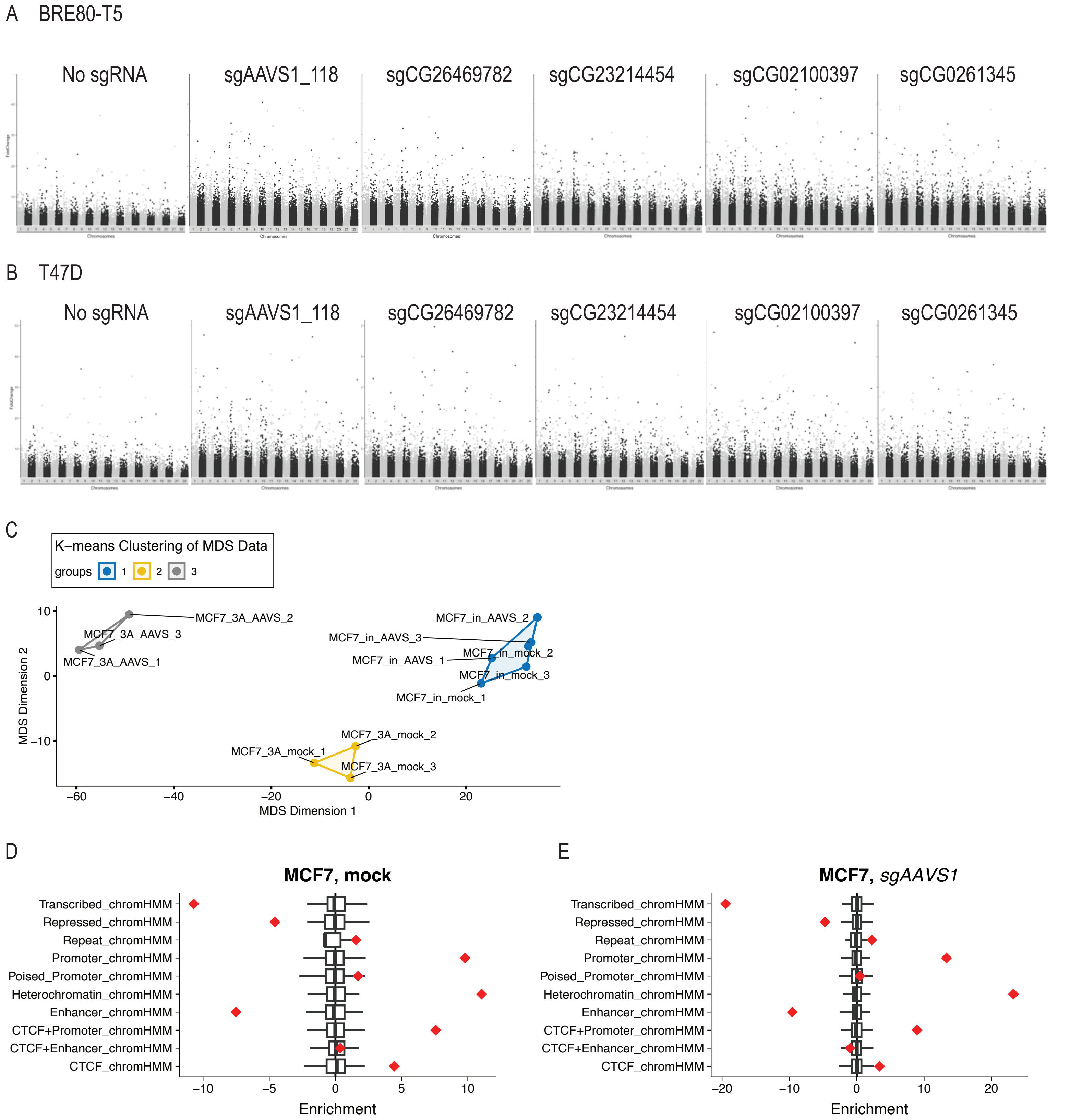


**Supplementary Figure 3: SAM-DNMT3A induces global non-specific DNA methylation.** (A) Manhattan plot showing fold changes in DNA methylation obtained by comparing DNA methylation levels (from EPIC arrays) in cells expressing the same sgRNA with SAM-DNMT3A and SAM-DNMT3A-inactive from with different sgRNA treatments in BRE80-T5. (B) Like (A) in T47D cells. (C) MDS dimensionality plot using all replicates of EPIC 1.0 array for MCF7 cells transduced with mock (control), SAM-DNMT3A-inactive or SAM-DNMT3A-active with sgAAVS1_118. (D) Enrichment of genomic features in MCF7 cells without an sgRNA. Random methylation patterns (black box and whiskers) were calculated by 100 random sampling of a similar sized methylation pattern. Enriched methylation sites are shown by red diamonds. Box limits indicate upper and lower quartiles and whiskers are 1.5 times of the minimum and maximum values, centre lines show the median. (E) Enrichment of genomic features in MCF7 cells with an AAVS1 targeting sgRNA. Random methylation patterns (black box and whiskers) were calculated by 100 random sampling of a similar sized methylation pattern. Enriched methylation sites are shown by red diamonds. Box limits indicate upper and lower quartiles and whiskers are 1.5 times of the minimum and maximum values, centre lines show the median.

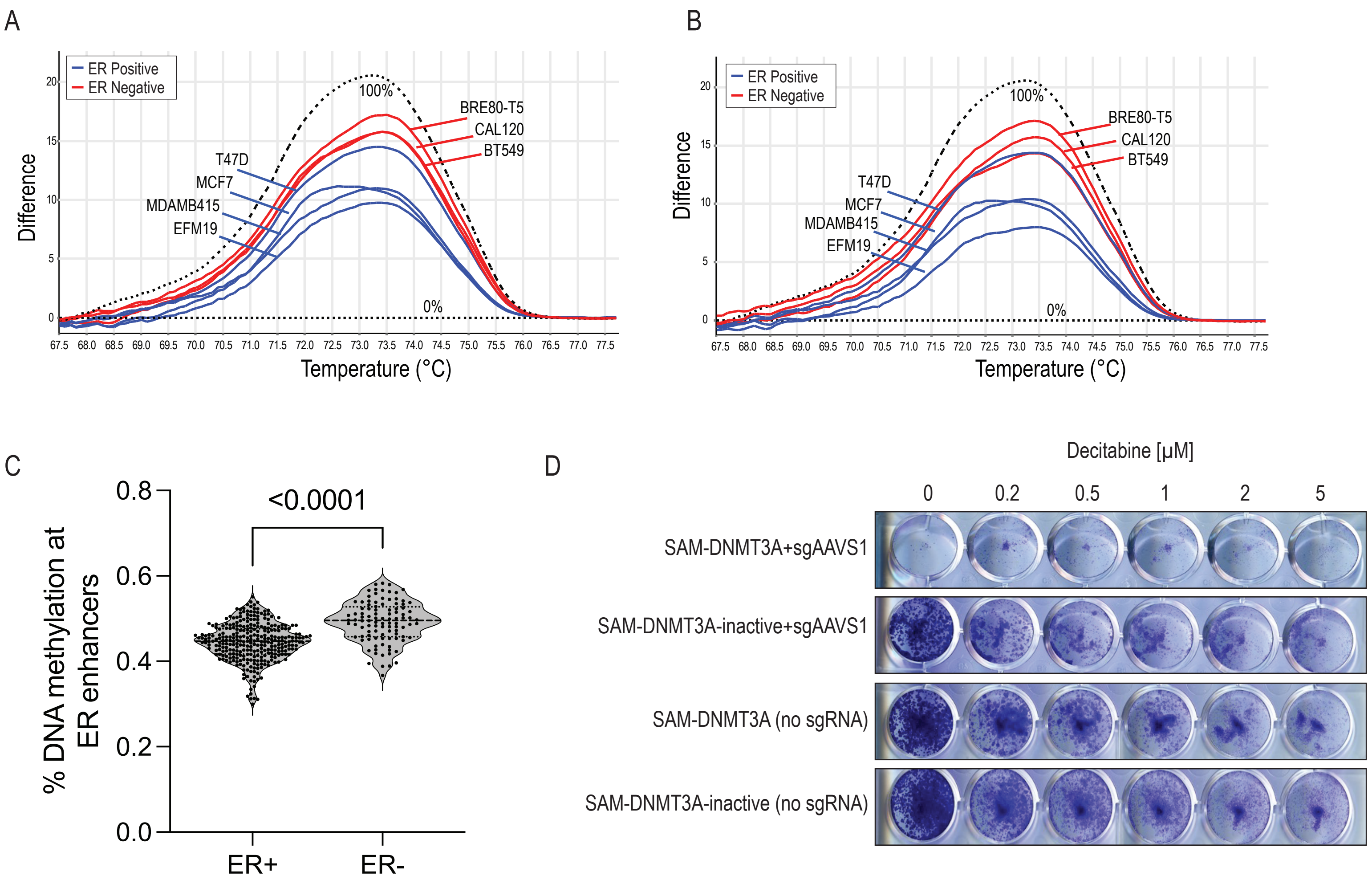


**Supplementary Figure 4: Induction of DNA methylation is a vulnerability in ER positive breast cancers.** (A,B) HRM assay quantifying DNA methylation at LINE1 sites. Dotted lines are standards containing the indicated percent of methylated DNA made from a mix of methylation and unmethylated DNA. (C) Levels of DNA methylation at ER-enhancers[32] in a cohort of 401 breast cancer tumours[33] separated by ER status. pValue calculated using a two tailed balanced T.test. n=301 ER-positive n=100 ER-negative (P = 5.9e18). (D) Crystal violet images of MCF7 cells expressing SAM-DNMT3A or SAM-DNMT3A-inactive with or without an AAVS1 targeting sgRNA treated with increasing concentrations of the DNMT inhibitor decitabine.
