# Supplementary material for: SAM-DNMT3A, a strategy for induction of genome-wide DNA methylation, identifies DNA methylation as a vulnerability in ER-positive breast cancers
Source: Nat Commun. 2024 Dec 1;15:10449. doi: 10.1038/s41467-024-54824-8 (PMC11609277; doi:10.1038/s41467-024-54824-8)
Supplement: Supplementary file 2 — Description of Additional Supplementary Files [file 41467_2024_54824_MOESM2_ESM.pdf]

Description of Additional Supplementary Files

File Name: Supplementary Data 1
Description: sgRNAs used in SAM-DNMT3A screens.

File Name: Supplementary Data 2
Description: Raw reads sgRNA and gene fold changes in pooled SAM-DNMT3A screens.

File Name: Supplementary Data 3
Description: Primers and sgRNA sequences used in this study.

File Name: Supplementary Movie 1
Description: Dynamics of SAM-DNMT3A with no sgRNA. Movie showing the dynamics of HaloTag-SAM-DNMT3A in HeLa cells expressing SAM-DNMT3A and no sgRNA.

File Name: Supplementary Movie 2
Description: Dynamics of SAM-DNMT3A with an sgRNA. Movie showing the dynamics of HaloTag-SAM-DNMT3A in HeLa cells expressing SAM-DNMT3A and an AAVS1 sgRNA.
