# Supplementary material for: SAM-DNMT3A, a strategy for induction of genome-wide DNA methylation, identifies DNA methylation as a vulnerability in ER-positive breast cancers
Source: Nat Commun. 2024 Dec 1;15:10449. doi: 10.1038/s41467-024-54824-8 (PMC11609277; doi:10.1038/s41467-024-54824-8)
Supplement: Supplementary file 8 — Reporting Summary [file 41467_2024_54824_MOESM8_ESM.pdf]

nature portfolio

Corresponding author(s): Joseph Rosenbluh and Melissa Southey

Last updated by author(s): Oct 16, 2024

Reporting Summary

Nature Portfolio wishes to improve the reproducibility of the work that we publish. This form provides structure for consistency and transparency in reporting. For further information on Nature Portfolio policies, see our Editorial Policies and the Editorial Policy Checklist.

Statistics

For all statistical analyses, confirm that the following items are present in the figure legend, table legend, main text, or Methods section.

n/a	Confirmed	
☐	☒	The exact sample size (*n*) for each experimental group/condition, given as a discrete number and unit of measurement
☐	☒	A statement on whether measurements were taken from distinct samples or whether the same sample was measured repeatedly
☐	☒	The statistical test(s) used AND whether they are one- or two-sided<br>*Only common tests should be described solely by name; describe more complex techniques in the Methods section.*
☐	☒	A description of all covariates tested
☐	☒	A description of any assumptions or corrections, such as tests of normality and adjustment for multiple comparisons
☐	☒	A full description of the statistical parameters including central tendency (e.g. means) or other basic estimates (e.g. regression coefficient) AND variation (e.g. standard deviation) or associated estimates of uncertainty (e.g. confidence intervals)
☐	☒	For null hypothesis testing, the test statistic (e.g. *F*, *t*, *r*) with confidence intervals, effect sizes, degrees of freedom and *P* value noted<br>*Give P values as exact values whenever suitable.*
☒	☐	For Bayesian analysis, information on the choice of priors and Markov chain Monte Carlo settings
☒	☐	For hierarchical and complex designs, identification of the appropriate level for tests and full reporting of outcomes
☐	☒	Estimates of effect sizes (e.g. Cohen's *d*, Pearson's *r*), indicating how they were calculated

Our web collection on statistics for biologists contains articles on many of the points above.

Software and code

Policy information about availability of computer code

Data collection	All software used is publicly available and provided in the methods section of the manuscript.
Data analysis	Data analysis was done using existing pipelines that include:<br>poolq - Used for alignment and quantification of CRISPR screen<br>MAGeCK - Used for CRISPR scoring<br>Prism - used for statistical analysis and plotting<br>R the minfi package -used for analysis of EPIC arrays<br>Thermo-Fisher HRM software - used for analysis of HRM data<br>MATLAB - used for analysis of Single Particle Tracking

For manuscripts utilizing custom algorithms or software that are central to the research but not yet described in published literature, software must be made available to editors and reviewers. We strongly encourage code deposition in a community repository (e.g. GitHub). See the Nature Portfolio guidelines for submitting code & software for further information.

Data

Policy information about availability of data

All manuscripts must include a data availability statement. This statement should provide the following information, where applicable:

- Accession codes, unique identifiers, or web links for publicly available datasets
- A description of any restrictions on data availability
- For clinical datasets or third party data, please ensure that the statement adheres to our policy

All raw and processed data are included in the supplementary tables or deposited in public databases

CRISPR screening processed and raw data is included in Supplementary Table 2
EPIC arrays are available through GEO (GEO accession number: GSE249125)

Research involving human participants, their data, or biological material

Policy information about studies with human participants or human data. See also policy information about sex, gender (identity/presentation), and sexual orientation and race, ethnicity and racism.

Reporting on sex and gender	Data presented describes analysis of DNA methylation in breast cancer and is restricted to females
Reporting on race, ethnicity, or other socially relevant groupings	The data collected is from a panel of 401 breast cancers. This dataset is from Melbourne Collaborative Cohort Study and is mostly white European origin women (described in detail in PMID: 28641380). No ethnicity was removed.
Population characteristics	This is a mixed cohort mostly 50-69 years old
Recruitment	The majority of participants were identified from the Victorian Electoral Enrolment Register (enrolment to vote is compulsory for all adult Australian citizens) and the Melbourne metropolitan phone directory, and were invited by letter to participate.
Ethics oversight	Monash University

Note that full information on the approval of the study protocol must also be provided in the manuscript.

Field-specific reporting

Please select the one below that is the best fit for your research. If you are not sure, read the appropriate sections before making your selection.

☒ Life sciences ☐ Behavioural & social sciences ☐ Ecological, evolutionary & environmental sciences

For a reference copy of the document with all sections, see nature.com/documents/nr-reporting-summary-flat.pdf

Life sciences study design

All studies must disclose on these points even when the disclosure is negative.

Sample size	For CRISPR screens we selected three cell lines that represent diversity in breast cancer.
Data exclusions	No data was excluded and all raw and processed data is presented
Replication	Screens were done in duplicates. Most experiments were done in triplicate (biological replicates). EPIC array experiment were done either 1 replicate or three replicate experiments (described in detail in legend).
Randomization	N/A
Blinding	N/A

Reporting for specific materials, systems and methods

We require information from authors about some types of materials, experimental systems and methods used in many studies. Here, indicate whether each material, system or method listed is relevant to your study. If you are not sure if a list item applies to your research, read the appropriate section before selecting a response.

Materials & experimental systems

n/a	Involved in the study
☒	☐ Antibodies
☐	☒ Eukaryotic cell lines
☒	☐ Palaeontology and archaeology
☒	☐ Animals and other organisms
☒	☐ Clinical data
☒	☐ Dual use research of concern
☒	☐ Plants

Methods

n/a	Involved in the study
☒	☐ ChIP-seq
☒	☐ Flow cytometry
☒	☐ MRI-based neuroimaging

Eukaryotic cell lines

Policy information about cell lines and Sex and Gender in Research

Cell line source(s)	MCF7 and T47D cells were a gift from Prof. Georgia Chenevix-Trench (QIMR-Berghofer).<br>HEK293FT cells were from Thermo-Fisher (#R70007).<br>BRE80-T5 cells were a gift from Prof. Roger Reddel<br>CAL120, BT549, EFM19 and MDAMB415 were from ATCC
Authentication	Cell-line identity was verified by STR at the Australian Genomics Research Facility (AGRF).
Mycoplasma contamination	All cell lines were regularly tested and found negative for mycoplasma using a PCR assay
Commonly misidentified lines<br>(See ICLAC register)	None of the cell lines we used are commonly misidentified lines

Plants

Seed stocks	*Report on the source of all seed stocks or other plant material used. If applicable, state the seed stock centre and catalogue number. If plant specimens were collected from the field, describe the collection location, date and sampling procedures.*
Novel plant genotypes	*Describe the methods by which all novel plant genotypes were produced. This includes those generated by transgenic approaches, gene editing, chemical/radiation-based mutagenesis and hybridization. For transgenic lines, describe the transformation method, the number of independent lines analyzed and the generation upon which experiments were performed. For gene-edited lines, describe the editor used, the endogenous sequence targeted for editing, the targeting guide RNA sequence (if applicable) and how the editor was applied.*
Authentication	*Describe any authentication procedures for each seed stock used or novel genotype generated. Describe any experiments used to assess the effect of a mutation and, where applicable, how potential secondary effects (e.g. second site T-DNA insertions, mosiacism, off-target gene editing) were examined.*
